# Potential application of waste from castor bean (*Ricinus communis* L.) for production for xylanase of interest in the industry

**DOI:** 10.1007/s13205-016-0463-1

**Published:** 2016-06-23

**Authors:** Polyanna Nunes Herculano, Keila Aparecida Moreira, Raquel Pedrosa Bezerra, Tatiana Souza Porto, Cristina Maria de Souza-Motta, Ana Lúcia Figueiredo Porto

**Affiliations:** 1 Mycology Department, Federal University of Pernambuco, Recife, PE Brazil; 2 Animal Morphology and Physiology Department, Federal Rural University of Pernambuco, Av. Dom Manoel de Medeiros s/n, Recife, PE 52171-900 Brazil; 3 Academic Unit of Garanhuns, Federal Rural University of Pernambuco, Garanhuns, PE Brazil

**Keywords:** Xylanase, Production, Solid-state fermentation, Aqueous two phase systems, *Aspergillus japonicus*

## Abstract

Xylanases activity (XY) from *Aspergillus japonicus* URM5620 produced by Solid-State Fermentation (SSF) of castor press cake (*Ricinus communis*) on different conditions of production and extraction by PEG/citrate aqueous two-phase system (ATPS) were investigated. XY production was influenced by substrate amount (5–10 g), initial moisture (15–35 %), pH (4.0–6.0) and temperature (25–35 °C), obtaining the maximum activity of 29,085 ± 1808 U g ds^−1^ using 5.0 g of substrate with initial moisture of 15 % at 25 °C and pH 6.0, after 120 h of fermentation. The influence of PEG molar mass (1000–8000 g mol^−1^), phase concentrations (PEG 20.0–24.0 % w/w and sodium citrate 15–20 % w/w) and pH (6.0–8.0) on partition coefficient, purification factor, yield and selectivity of XY were determinate. Enzyme partitioning into the PEG rich phase was favored by *M*_PEG_ 8000 (g mol^−1^), *C*_PEG_ 24 % (w/w), *C*_C_ 20 % (w/w) and pH 8.0, resulting in partition coefficient of 50.78, activity yield of 268 %, 7.20-fold purification factor and selectivity of 293. *A. japonicus* URM5620 has a potential role in the development of a bioprocess for the XY production using low-cost media. In addition, the present study proved it is feasible to extract xylanase from SSF by adopting the one step ATPS consisting of PEG/citrate.

## Introduction

With the increasing concern to valorize the waste material constantly generated, there is considerable interest in the establishment of efficient processes to obtain valuable products from residues that permit the recovery of commercially attractive products from a wide variety of residues (Soccol et al. 2011). In addition, the growing bioprocess industry depends on the progressive reduction of expensive nutrient inputs into fermentation media. The potential production and recovery of enzymes from microbial using cheap agricultural by-products such as castor press cake (*Ricinus communis*) improve the commercial feasibility of bioprocess technology. About 1 Mt of castor beans is harvested annually for castor oil production, with India, China, and Brazil being major producers (Mutlu and Meier 2010).

Xylan is the second most abundant polysaccharide in nature and it is the major hemicellulosic polysaccharide of wood and agricultural wastes, where it comprises up to 20–35 % dry weight. Considering the nutritional composition of castor press cake, commercial applications for such a valuable material, hitherto, considered a waste in most developing countries need to be explored and one such application could be their use as a substrate for xylanase enzyme production. Xylanase (XY) is the main enzyme which degrades linear polysaccharide *β*-1,4-xylan into xylose, thus breaking down hemicelluloses (Irfan et al. 2014). The design of efficient production and recovery protocols that allow the use of raw material to enzymes production is one of the key goals in the field.

High production cost and low production yields had caused the bottleneck for industrial enzyme applications, thus alternate enzyme production method using cheaper ingredient with higher yield is necessary. Several works using different substrates has been made to increase enzyme production and reduce production costs (Ang et al. 2013; Pandya and Gupta 2012). SSF is advantageous since it has greater volumetric productivities, higher product stability, low contamination risk and lower instrumental costs. Another advantage is the use of cheap solid agro-lignocellulose wastes which acts as carbon and energy source and further reduce the need of expensive nutrient medium. *Aspergillus* sp. was commonly classified as strong xylanase producer (Collins et al. 2005).

*Aspergillus japonicus* URM5620 cultivated in the SSF with castor bean meal as carbon source produces (hemi) cellulase enzymes in a shorter period with cheap and available raw material (Herculano et al. 2012). Particularly, *Aspergillus* sp. are considered representative model to production of commercial interest substances (Khammuang and Sarnthima 2007). The world market of xylanase is expanding speedily due to its enormous pivotal roles in various industries, particularly in the biotechnology applications, including pulp and paper, baking, animal feed, detergent, food and beverage (Ho and Lau 2014).

The downstream processing (DSP) of biomolecules is often representing a major bottleneck of the whole production process in terms of complexity and high cost, which can make up more than 70 % of the total DSP product cost (Raja et al. 2011). In this context, aqueous two-phase system (ATPS) is a promising downstream processing to sensitive biomolecules and biotechnological products (Glyk et al. 2015). PEG/salt characteristics have been exploited for several biomolecules extraction by to be primary recovery technique that integrates the concentration and partial purification of important biomolecules from their natural source in a single step (Rito-Palomares and Lyddiatt 2002).

In this context, the purpose of this study was to evaluate the production of xylanase (XY) from *A. japonicus* URM5620 by Solid-State Fermentation (SSF) of castor press cake (*Ricinus communis*) and exploits the partition and purification of xylanase in ATPS made up of PEG/citrate.

## Materials and methods

### Castor bean meal

The castor bean meal used in this study was supplied by the Brazilian Agricultural Research Corporation, EMBRAPA/Agribusiness Tropical, located in Fortaleza, Ceará, Brazil, which is currently developing a Programme of Development of Castor in the State (Rodrigues 2007; Oliveira et al. 2013).

### Microorganism

The culture *A*. *japonicus* URM5620 utilized in this study was obtained from the Mycology Department’s Mycoteca—URM, at Federal University of Pernambuco, Brazil. The strain was maintained on Malt Extract agar and kept at 28 °C for 7 days.

### Xylanase production (XY) by SSF

For XY production, castor cake was used as the substrate with a particle size between 3 and 8 mm to provide improved absorption and porosity to facilitate transport of oxygen and nutrients during SSF (Spier et al. 2008). The substrate was autoclaved at 120 °C for 15 min in Erlenmeyer flasks of 250 mL. The inoculum was prepared by suspending the spores present on the malt extract agar plates in 0.05 M citrate buffer. The number of spores was determined in a Neubauer counting chamber, and the inoculum of 10^7^ spores per gram of dry weight was inoculated in the substrate used for SSF. The initial moisture of the substrate was determined in accordance with the standards of the Instituto Adolfo Lutz (2005). Substrate was dried at 105 °C for until constant weight and 100 % moisturization was achieved by adding of distilled water on substrate. Dry solid substrate was mixed with predetermined amount of distilled water until achieve required initial moisture.

### Preparation of the enzymatic extract

The XY production was followed for 120 h. The contents of the flasks were harvested at regular intervals (24 h) by adding 0.05 M citrate buffer, (each 1 g of substrate: 2.5 mL of buffer), incubated in a temperature controlled bath at 32 °C for a period of 1 h and filtered with filter paper (Whatman No. 1) under vacuum. The supernatant was used as enzymatic extract crude and subjected to enzymatic analysis (Herculano et al. 2011).

### Preparation of aqueous two-phase systems

Aqueous two phase systems (ATPS) were prepared in 15 mL graduated tubes by mixing appropriate amounts of 50 % w/w stock solutions of different molecular weights PEG (1000, 3350 and 8000 g mol^−1^), 50 % w/w stock solution of potassium citrate, at different pH values (6.0, 7.0, 8.0), at 25 ± 1 °C, according to statistical design described in the Table 2. Fermentation broth representing (20 % w/w) and water and was added to a final weight of 5 g. After vortex shaking for 1.0 min, the two phases were separated by settling for 40 min. Then, the phase volumes were measured; top and bottom phases were separately withdrawn with pipettes and assayed for protein concentration and xylanase activity.

### Analytical techniques

Protein content was determined according to Bradford (1976) at 595 nm. Bovine serum albumin was used as a standard. XY activity was determined according to the methodology described by Bailey et al. (1992). The activity was carried out in the total reaction mixture of 1 mL containing 0.1 mL of suitably diluted enzyme and 0.9 mL of 1 % (w/v) of xylan (Sigma, USA) solution in sodium citrate buffer (50 mM, pH 5.0 at 60 °C). This reaction mixture was incubated at 50 °C for 5 min. One unit (U) of enzyme activity was defined as the amount of enzyme required to liberate 1 µmol of xylose (Vetec, Brazil) from the appropriate substrates per minute under the assay conditions. The release of reducing sugars was determined by the 3,5-dinitrosalicylic acid (DNS) method Miller (1959).

### Experimental design and statistical analysis

The experiments performed according to a 2^4^ full factorial design with three levels plus four central points for xylanase production (Table 1) and xylanase extraction using ATPS systems (Table 2). The influence of the substrate amount (*Sa;* 5–10 g), initial moisture (*Im,* 15–35 %), temperature (*T,* 25–35 °C*)* and pH (4.0–6.0) on the XY production was evaluated (Table 1). The choice of variables and their levels was made according to Gao et al. (2008). In the best production condition, the XY was extracted using poly (ethylene glycol)–sodium citrate aqueous two-phase systems (ATPS). The influence of variables, namely PEG molar mass (*M*_PEG_), PEG concentration (*C*_PEG_), citrate concentration (*C*_C_) and pH was studied on the three selected responses: purification factor (PF), activity yield (*Y*), partition coefficient (*K*) and selectivity (*S*) (Table 2). The significance of effects and two factor interactions was estimated by ANOVA. The main effect of the independent variables and their interactions on the dependent variables was investigated using Pareto chart and interaction plot. All statistical analyses were carried out using *Statistica* 8.0 software (StatSoft Inc., Tulsa, OK, USA).

**Table 1:** Full factorial design for xylanase production (XY) in solid-state fermentation (SSF) by *Aspergillus japonicus* URM5620 under different operational conditions after 120 h of cultivation

Run	Sa (g)^a^	Im (%)^b^	pH^c^	T (°C)^d^	XY	
					(U g ds^−1^)^e^	(U mg^−1^)^f^
1	5.0	15	4.0	25	8200	29,491
2	5.0	15	4.0	35	401	1810
3	5.0	35	4.0	25	8967	24,128
4	5.0	35	4.0	35	8898	24,378
5	10.0	15	4.0	25	4217	19,027
6	10.0	15	4.0	35	5517	21,130
7	10.0	35	4.0	25	4455	12,367
8	10.0	35	4.0	35	2659	7880
9	5.0	15	6.0	25	29,085	62,141
10	5.0	15	6.0	35	2766	6256
11	5.0	35	6.0	25	7664	17,032
12	5.0	35	6.0	35	3585	8883
13	10.0	15	6.0	25	4188	11,243
14	10.0	15	6.0	35	7452	20,573
15	10.0	35	6.0	25	4137	9540
16	10.0	35	6.0	35	3075	7901
17	7.5	25	5.0	30	5661	16,162
18	7.5	25	5.0	30	7479	22,819
19	7.5	25	5.0	30	6723	17,590
20	7.5	25	5.0	30	6387	17,381

^a^ Sa-substrate amount (g, grams), ^b^ Im-initial moisture (%), ^c^ pH and ^d^ T-temperature (°C), ^e^ U gds^−1^—xylanase activity, ^f^ U mg^−1^—specific xylanase activity

**Table 2:** Effects of PEG molar mass (*M*_PEG_), PEG concentration (*C*_PEG_), citrate concentration (*C*_C_) and pH on partition coefficient (*K*), top activity yield (*Y*), purification factor in the top phase (*PF*), selectivity (*S*) using 2^4^ experimental design to xylanase extraction by PEG/citrate ATPS

Run	*M*_PEG_^a^	*C*_PEG_^b^	*C*c^c^	pH	*K* ^d^	*Y* ^e^	PF^f^	S^g^
A1	1000	20	15	6.0	27.20	268.01	7.16	185.25
A2	8000	20	15	6.0	9.66	183.03	6.05	83.04
A3	1000	24	15	6.0	35.98	265.12	5.36	208.14
A4	8000	24	15	6.0	19.28	220.00	4.00	95.91
A5	1000	20	20	6.0	47.15	192.73	3.27	146.06
A6	8000	20	20	6.0	31.41	163.59	1.13	5.52
A7	1000	24	20	6.0	38.49	184.92	3.66	138.71
A8	8000	24	20	6.0	44.44	179.17	4.88	293.33
A9	1000	20	15	8.0	45.27	229.94	6.51	286.69
A10	8000	20	15	8.0	24.39	198.47	6.68	218.78
A11	1000	24	15	8.0	28.68	205.94	3.38	105.20
A12	8000	24	15	8.0	20.62	192.35	2.44	67.42
A13	1000	20	20	8.0	37.87	160.77	3.94	135.58
A14	8000	20	20	8.0	28.10	181.54	4.65	128.86
A15	1000	24	20	8.0	30.23	154.14	2.05	67.48
A16	8000	24	20	8.0	50.78	207.08	2.50	108.60
A17	3350	22	17.5	7.0	24.31	207.10	7.20	203.35
A18	3350	22	17.5	7.0	21.56	184.27	4.91	141.84
A19	3350	22	17.5	7.0	22.86	199.04	5.58	152.38
A20	3350	22	17.5	7.0	28.96	239.34	5.81	231.20

^a^PEG molar mass (g mol^−1^), ^b^ PEG concentration (%), ^c^ citrate concentration (%), ^d^ partition coefficient, ^e^ top activity yield (%), ^f^ purification factor in the top phase, ^g^ selectivity

### Determination of partition coefficient, purification factor, yield and selectivity

The XY partition coefficient was defined as the ratio of the volumetric activity in the top phase (*A*_t_) to that in the bottom phase (*A*_b_):

1 $$K = \frac{{A_{\text{t}} }}{{A_{\text{b}} }}$$

The purification factor was calculated as the ratio of the specific activity in the top or bottom phase to the specific activity in the cell extract before partition (*A*_i_):

2 $$\text{PF} = \frac{{A_{\text{x}} /C_{\text{x}} }}{{A_{\text{i}} /C_{\text{i}} }}$$

where *C*_x_ and *C*_i_ are the total protein concentrations, expressed as µg mL^−1^, in the top or bottom phase and initial extract, respectively.

The activity yield was determined as the ratio of total activity in the top or bottom phase to that in initial extract and expressed as percentage:

3 $$Y = \left( {\frac{{A_{\text{x}} .V_{\text{x}} }}{{A_{\text{i}} .V_{\text{i}} }}} \right) \times 100$$

where *V*_x_ and *V*_i_ are the volumes of the top or bottom phase and the initial extract, respectively.

The selectivity was defined as the ratio of the enzyme partition coefficient (*K*_e_) to the protein partition coefficient (*K*_p_) (Mayerhoff et al. 2004):

4 $$S = \frac{{K_{\text{e}} }}{{K_{\text{p}} }}$$

## Results

The results obtained from the 20 runs of the first experimental design are showed in Table 1. Fermentation time in all runs was 120 h which obtained the highest xylanase activities.

**Fig. 1:**
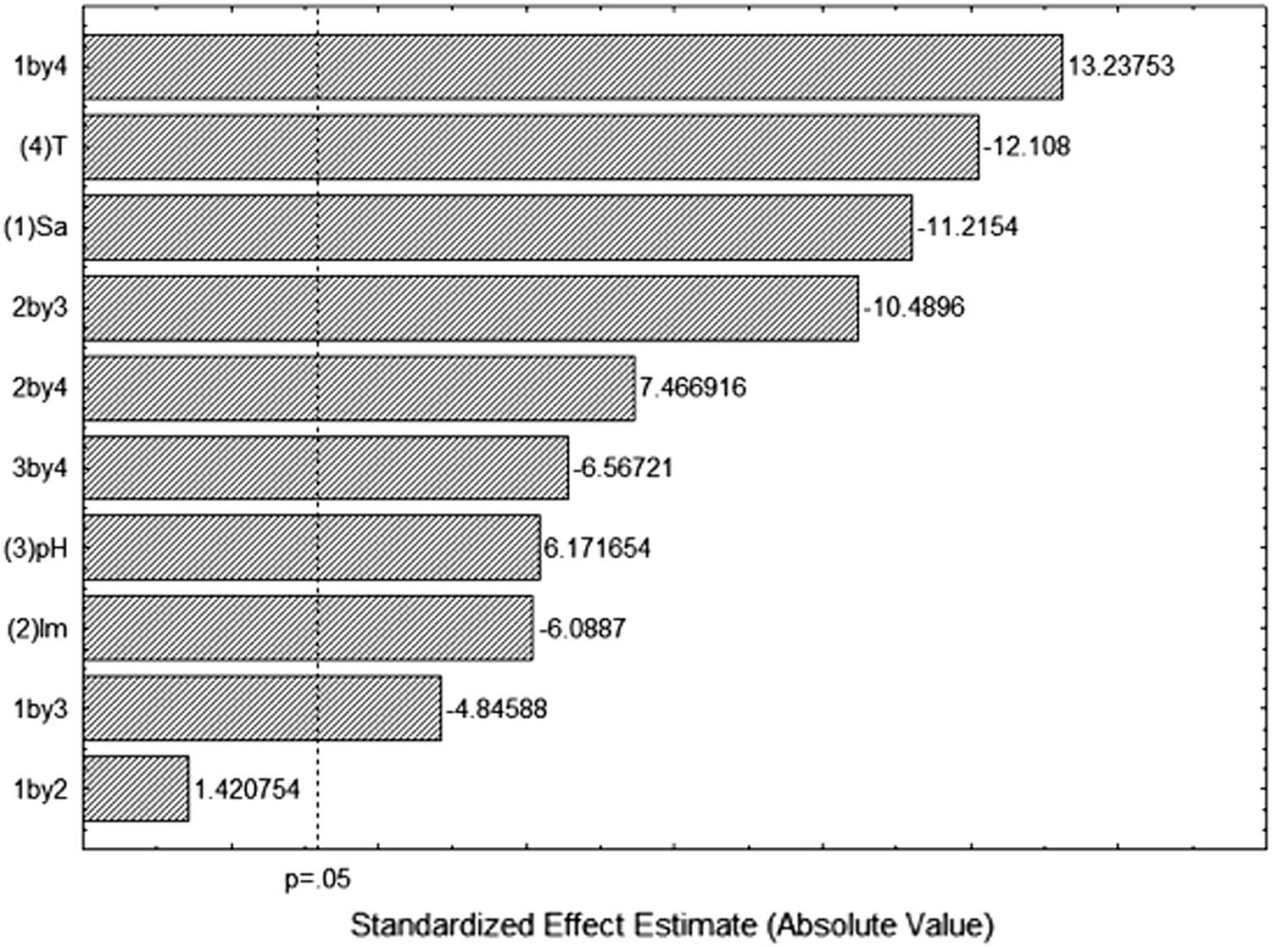
Pareto chart for the standardized effects of the variables: (1) temperature, (2) initial moisture, (3) substrate amount and (4) pH on activity of xylanase after 120 h of fermentation

The effects of variables studied on xylanase activity were shown in Pareto Chart (Fig. 1). All principal variables and their interaction showed a significant response effect (*p* < 0.05), except the interaction between *Sa* and *Im*. The pH showed a positive and significant effect, while that increasing *Im*, *Sa* and temperature reduces the xylanase production (Fig. 1). Xylanase production increases with increasing pH. On the other hand, lower amount substrate, initial moisture and temperature increased XY by *A. japonicus* URM5620 in Solid-State Fermentation (SSF) of castor press cake (*Ricinus communis*). Maximum XY activity of 29,085 ± 1808 U g ds^−1^ was produced in the culture with 120 h of fermentation, using 5.0 g of substrate with initial moisture of 15 % at 25 °C and pH 6.0 (run 9; Table 1).

**Fig. 2:**
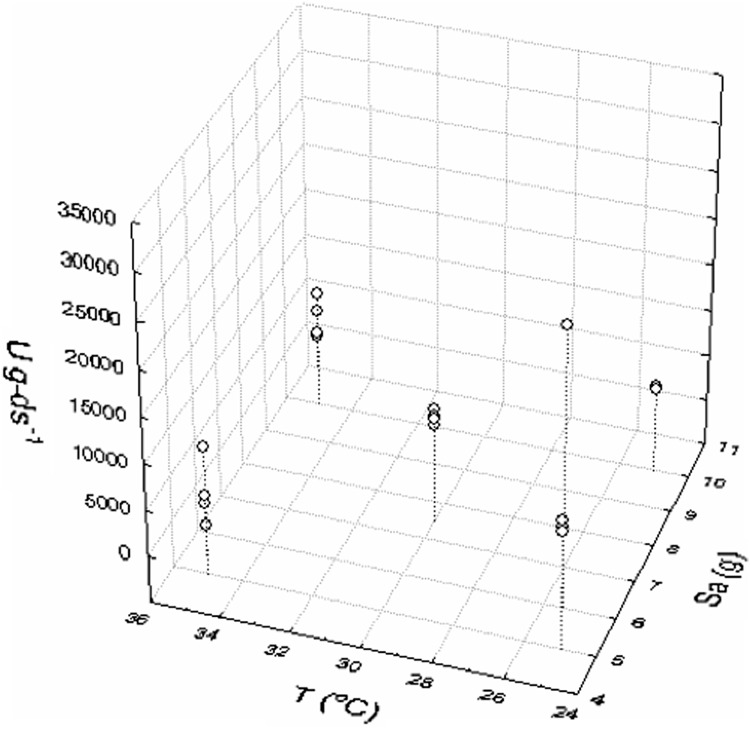
Scatterplot of the *Sa* and *T* effects on the activity of xylanase (XY, U g ds^−1^)

Figure 1 shows that temperature and substrate amount were two variables most significant in the whole set of experiments to xylanase production. Therefore, they were selected for scatter plot showing the highest XY activity at 25 °C and pH 6.0 (Fig. 2).

In the best production condition, the enzymatic extract was subjected to ATPS technique using PEG/citrate to extract and/or purify xylanase. ATPS can be effectively used in concentrating of xylanases from the crude enzymes. Table 2 shows *K* values were higher than 1 in all runs, indicating the effectiveness of xylanase partitioning to the top phase. The highest value of *K* was 50.7 (Run A16; Table 2).

Table 3 shows the effects of the independent variables and of each combination on dependent variables *K*, *Y*, PF and *S*. *M*_PEG_ and *C*_C_ had influence on *K* value (*p* < 0.05; Table 3). *M*_PEG_ have a negative effect indicating that lower molecular weight increase the *K*, pushing enzyme partition towards the top phase. Xylanase enzymes presents higher affinity for the PEG-rich phase, especially when PEG of low molecular weight was utilized. *C*_C_ have positive effect, indicating that lower concentrations increase the *K*. Xylanase partition has a tendency to shift to the top phase due to salting-out effects caused by high *C*_C_ that generate the movement of the enzyme to this phase. On the other hand, *C*_PEG_ e pH not influenced statistically on *K* values.

**Table 3:** Effects estimates of the responses obtained of the 2^4^-experimental design for partition coefficient (*K*), top activity yield (*Y*), purification factor in the top phase (*PF*), selectivity (*S*) of xylanase extraction by PEG/citrate ATPS

	*K*	*Y*	PF	*S*
1—*M*_PEG_	−4.81*	−1.46	−2.60	−1.60
2—*C*_PEG_	1.35	0.32	−7.18*	−0.62
3—*C*_C_	7.54*	−3.64*	−10.0*	−1.33
4—pH	0.95	−1.35	−2.17	−0.22
1*2	5.08*	1.21	1.12	2.14
1*3	4.97*	2.29	2.24	2.17
1*4	2.00	2.08	2.44	0.76
2*3	1.65	0.24	7.31*	2.89
2*4	−2.17	−0.56	−7.56*	−4.35*
3*4	−3.20	0.99	2.43	−1.47

* Statistically significant value (at the 95 % confidence level, *p* < 0.05)

The value of enzymatic yield (*Y*) on top phase varied between 154 % in system A15 and 268 % in system A1 (Table 2). The main effects of variables on the activity recovery in top phase of PEG–citrate systems and their significance are shown in Table 3. *C*_C_ influenced negatively and statistically in the *Y* values, showing the increase in the *Y* with lower *C*_C_ (15 %) while the variables *M*_PEG_, *C*_PEG_, pH and the variable interaction not influenced in the *Y* values (Table 3).

**Fig. 3:**
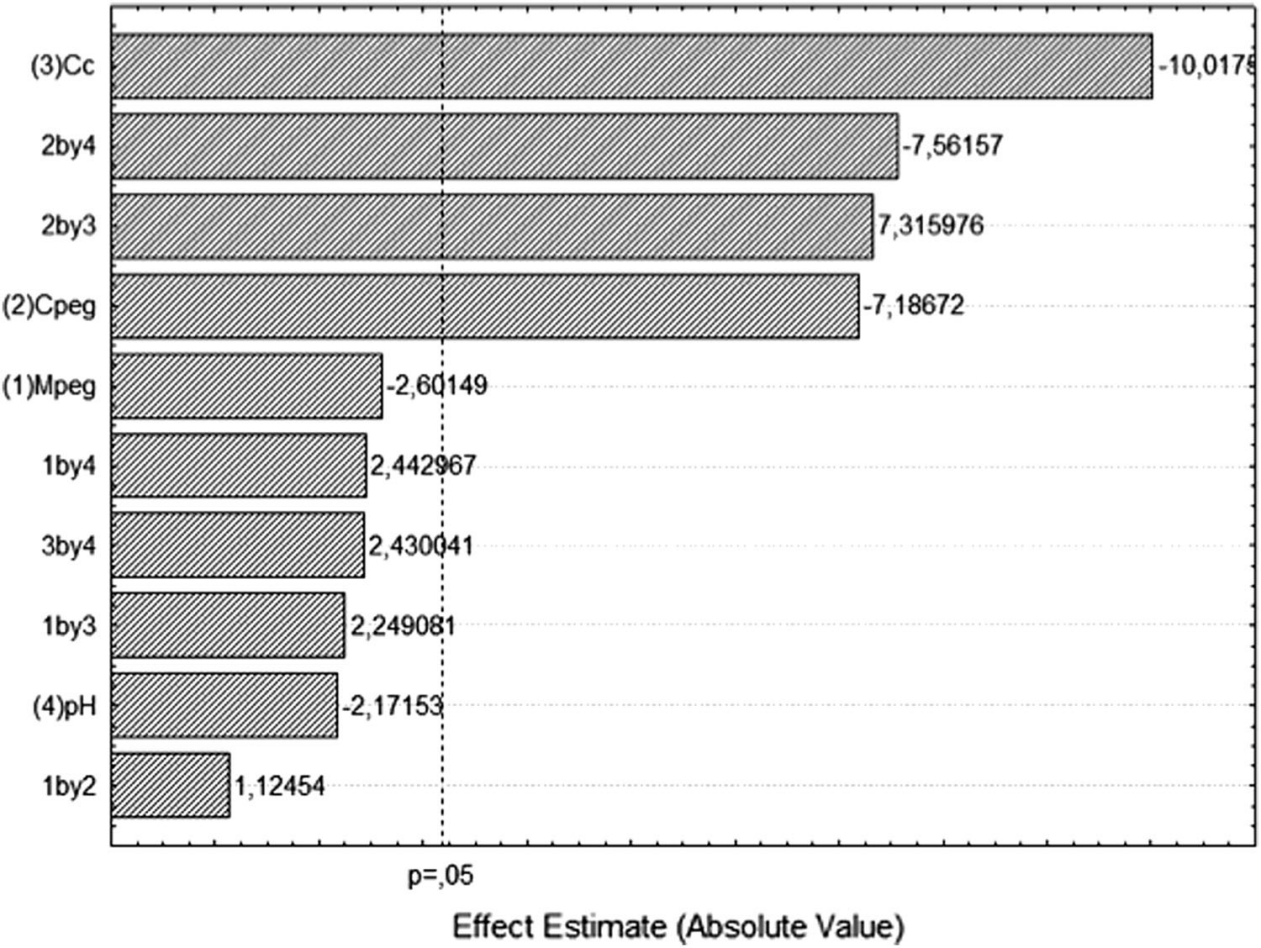
Pareto chart for the standardized effects of the variables: (1) PEG molar mass—*M*_PEG_, (2) PEG concentration—*C*_PEG_, (3) citrate concentration —*C*_C_ and (4) pH on purification factor

*PF* in top phase varied between 1.13 in A6 system and 7.81 in A20 system (Table 2). The main effect of variables *M*_PEG_, *C*_PEG_, *C*_C_ and their interaction on PF can be observed in. Table 3. *C*_PEG_, *C*_C_ and interaction between *C*_PEG_ e pH had negative effects on the PF while *C*_PEG_ and *C*_C_ interaction had positive effects on the response (Fig. 3). The negative interaction effects of *C*_PEG_ and pH means that an increase in *C*_PEG_ with a simultaneous decrease in pH increase PF. On the other hand, the positive interaction effect between *C*_PEG_ and *C*_C_ means that simultaneous increase of *C*_PEG_ and *C*_C_ results in higher *PF* and enzyme load gives more active enzyme in the top phase.

The selectivity of the systems had higher values (*S* = 293; Table 2) in systems employing *M*_PEG_ 8000 (g mol^−1^), *C*_PEG_ 24 % (w/w), *C*_C_ 20 % (w/w) and pH 6.0. The results of the statistical analysis with the effects of each variable studied in the experimental design to evaluate the selectivity of the systems are shown in Table 3. The only significant negative interaction between *C*_PEG_ and pH on the selectivity was also detected, which means that an increase in *C*_PEG_ and a simultaneous decrease in pH led to higher selectivity. PEG/Citrate ATPS system that has higher selectivity of extraction, obtained moderate *Y* and *PF* values (A8; Table 2) while that the systems with high *Y* and *PF* values obtained selectivity moderate (A1; Table 2). Therefore, ATPS could be used to selectively purify xylanase from the crude culture in a single-step operation.

## Discussion

Xylanase production by *A. japonicus* URM5620 in solid-state fermentation (SSF) of castor press cake (*Ricinus communis*) is influenced by variables substrate amount initial moisture, pH and temperature. High XY activities (29,085 ± 1808 U g ds^−1^) were obtained using castor bean meal by solid-state fermentation, denoting that this agroindustrial residue without any addition of nutrient solution is a good substrate for XY production by *A.* *japonicus*.

An increase in pH increases XY activity (Fig. 1). The enzyme production by microbial strains depends on the initial pH of the medium as it influences many enzymatic processes and transport of various components across the cell membrane (Prakasham et al. 2005). The present results show that xylanase production was higher with increasing pH values, obtained high value at pH 6.0. This is according to XY production (2012 ± 18 U g ds^−1^) produced by *Aspergillus tubingensis* FDHN1 in sorghum straw SSF (Adhyaru et al. 2015) and by of *Aspergillus tubingensis* JP-1 using untreated wheat straw (Pandya and Gupta 2012).

Extracellular xylanase production appeared to be sensitive to the initial moisture content of the castor press cake. An increase in the *Im* reduced xylanase production by *A. japonicus* URM5620 in Solid-State Fermentation (SSF) of castor press cake (*Ricinus communis*) (Fig. 1), obtained the highest value at 15 %. It showed that water presents several crucial roles in cell metabolism during SSF. The lower xylanase production at higher moisture levels could attribute to alteration in particle structure, decrease in porosity or lower oxygen transfer, whereas the lower moisture content leads to a reduction in the diffusion of the nutrients and oxygen in the substrate, lower degree of swelling and higher water tension (Hasseltine 1972). On the other hand, Soliman et al. (2012) related that 75 % was the optimum initial moisture for xylanase production by *A. niger* (21.32 U g^−1^) in using barley bran and Ang et al. (2015) related that initial moisture of 75–80 % is optimal to xylanase production using *Aspergillus fumigatus* SK1 cultivated SSF with in oil palm trunk.

The optimum moisture was closely depended on some other parameters such as; organism, desired product as well as nature of substrate (i.e., physical, chemical and their water-binding characteristics, in addition with particle size). Low moisture may reduce the solubility and swelling capacity of substrate causing high-water tension, decreasing growth and enzyme production (Poorna and Prema 2007). Another advantage is that low moisture level in the system by it be easier to control contamination. In contrast, high moisture adversely affects the microstructure between solid particles by creating a gummy texture. This cause the microscopic pores in the solid substrate and thus further interrupts the gas phase between particles, resulting in an anaerobic microenvironment (Chen 2013). Since fungi are known to thrive in moist environments, optimum moisture thus critically controls the success of their growth, as well as the production of metabolites. The moisture content in SSF is a crucial factor that determines the success of the process (Ramesh and Lonsane 1990).

Temperature is one the most significant factors that affects the production of enzyme. Xylanase production peaked at temperature of 25 °C, decreasing above this value. It was also observed by *A. niger* grown on *Jatropha* seed cake (Ncube et al. 2012) and *Aspergillus oryzae* P6B2 cultivated under SSF with using wheat bran (Pirota et al. 2013) The biosynthesis of xylanase by *A. oryzae* was highly sensitive to variations in temperature, indicating that this variable should be carefully monitored and controlled during the cultivation process. Temperature is important because affect microbial growth, metabolism, and spore germination. In addition, it provides energy for the enzymes to reach the transition state and, thus, induce the enzyme–substrate catalysis reaction (Lu et al. 2008). However, excess energy causes thermo denaturation of the enzymes, which is responsible for the metabolism, causing the fungus to synthesize only essential protein that is crucial for growth (Ogino et al. 2001). It has been shown in a previous study that *A. tubingensis* JP-1 was unable to produce a considerable xylanase yield above 30 °C (Pandya and Gupta 2012). On the other hand, Ang et al. (2015) related that maximum xylanase production by *Aspergillus fumigatus* SK1 cultivated SSF with in oil palm trunk was between 40 and 45 °C and Adhyaru et al. (2015) found the highest XY production of 1998 ± 12 U g ds^−1^ by *Aspergillus tubingensis* FDHN1 in sorghum straw SSF at 40 °C. It shows that the type of fermentable wastes and species of microorganisms may also affect the value of incubation temperature.

Xylanase production is also affected by the amount substrate. The effect negative of amount substrate on xylanase production indicates that increasing the substrate amount there are detrimental role on enzyme production in the present study (Fig. 1) shows that high concentration of castor press cake inhibits the enzyme synthesis. Castor press cake concentration mediated regulation of xylanase production in this fungal strain. Of all these tested concentrations of substrate 5 g showed improved enzyme production. Our findings were in accordance with Suvarna Lakshmi et al. (2009) who also reported that when increase the substrate level, it caused reduction of xylanase production by using *A. terreus* grown on rice straw in SSF.

In current research, *A. japonicus* URM5620 was a higher xylanase producer (29,085 ± 1808 U/g ds; Table 1) compared to *A. fumigatus* SK1 under SSF using untreated oil palm trunk (418.7 U g^−1^) and by *Aspergillus awamori* IOC-391 using babassu cake (835.0 U g^−1^; Castro et al. 2015).

PEG/citrate ATPS was effectively used to extract the extracellular xylanase produced by *A. japonicus* URM5620 from the crude enzymes. In this work, *K* values indicate the effectiveness of partitioning to the top phase (Mazzola et al. 2008). In the general, most of the purified xylanase activity by ATPS was collected in the top phase (Garai and Kumar 2013; Yang et al. 2008). Usually the partition coefficient decreases as the PEG chain length increases, behavior also observed in the present case for the proteins and xylanase enzymes. As the bio-molecule size increases, its preference for partitioning into one phase also tends to increase. Another aspect that should be taken into account is the relative hydrophobicity of the enzyme surface. For bio-molecules with similar size and net charge, a higher presence of hydrophobic regions at the molecule surface tends to enhance its preference for the polymer phase, an aspect that eventually can also explain the differences in the partitioning behavior of the xylanase enzymes observed in the present work. Xynalase from *Aspergillus candidus* exhibits a high preference for the top phase as expected for a hydrophobic enzyme as observed by Garai and Kumar (2013) using ATPS composed of PEG 4000/NaH_2_PO_4_ system.

The recovery of active enzyme and PF in the top phase decreased with increase in *C*_C_ (Table 2). This would result in salting out of proteins and increased partition to the top phase, providing a lower Y in this phase. Furthermore, PEG is known to refold the enzyme increase their activity. As mentioned, other reports have suggested that PEG promotes proper refolding, playing a similar function to that of chaperone proteins (Sánchez-Trasviña et al. 2015; Cleland and Wang 1990). This same effect was observed in xylanase from *Bacillus halodurans* in the PEG–phosphate two-phase systems (Rahimpour Mamo et al. 2007) and invertase from *S. cerevisiae* in PEG–potassium phosphate (Sánchez-Trasviña et al. 2015).

The highest value of *Y* (268 %) and *PF* (7.16) was obtained in the top phase systems composed by *M*_PEG_ 1000 (g mol^−1^), *C*_PEG_ 20 % (w/w), *C*_C_ 15 % (w/w) and pH 6.0, indication that xylanase was purification of contaminant proteins with high enzymatic yield. This promising result was due to xylanase partition to the two phases almost to the same extent, while the other proteins preferably partitioned to the bottom phase, providing to be an excellent system for purification of the xylanase in a single-step operation. Yang et al. (2008) extracted and purified xylanase one step in ATPS composed by PEG and (NH_4_)_2_SO_4_, obtaining the purity of the target xylanase is comparable to obtained from column chromatography. Thus, the ATPS is demonstrated to be efficient and inexpensive xylanase purification if the correct conditions are selected.

Values of the yield higher than 100 % have been reported for enzyme extraction using liquid–liquid (Porto et al. 2008; Cavalcanti et al. 2006). Sánchez-Trasviña et al. (2015) related that o PEG can refold the enzyme increase their activity. Pancera et al. (2000) reported that PEG can influence enzyme activity, because alteration of the structure of enzyme active site in the presence of PEG might enhance its relative activity. Mayerhoff et al. (2004) this behavior with the elimination of inhibitors from the PEG phase during the extraction as well as with the very composition of the system, which may favor the enzymatic activity.

This investigation has clearly established the effect of growth conditions on the production of xylanase by *Aspergillus japonicus* URM5620 showing it to be a potential microorganism for the XY using SSF. Temperature, pH, moisture and substrate amount were significant factors to enzymatic production. No addition of expensive media is required and the use of inexpensive agro-industrial wastes will have important economic advantages. In addition, we investigated the effects of environmental conditions on xylanase purification in PEG/citrate aqueous two phase systems ATPS, including the system composition, the PEG molecular weight, PEG concentration, Citrate concentration and pH values. Partial purification was attained, with a good compromise between the purification factor and the yield was achieved within the experimental domain investigated using a simple and inexpensive procedure. A purification factor of 7.16 and a yield as high as 268 % in top phase was obtained for Xylanase in the system containing *M*_PEG_ 1000 (g mol^−1^), *C*_PEG_ 20 % (w/w), *C*_C_ 15 % (w/w) and pH 6.0. These results demonstrate the feasibility of single-step a xylanase purification in PEG-citrate ATPS with low environmental toxicity.

Castor press cake can be used in SSF to xylanase production by *Aspergillus japonicus* URM5620 as well as this enzyme can be extracted by PEG/Citrate ATPS system.
